# Trafficking of *Mycobacterium tuberculosis* Envelope Components and Release Within Extracellular Vesicles: Host-Pathogen Interactions Beyond the Wall

**DOI:** 10.3389/fimmu.2020.01230

**Published:** 2020-07-16

**Authors:** Emilie Layre

**Affiliations:** Institut de Pharmacologie et de Biologie Structurale, Université de Toulouse, CNRS, Université Paul Sabatier, Toulouse, France

**Keywords:** lipids, tuberculosis, vesicles, exosomes, host-pathogen interactions

## Abstract

Components of *Mycobacterium tuberculosis* (Mtb) envelope such as lipoproteins, lypoglycans, lipids, and glycolipids act as Pathogen Associated Molecular Patterns and/or antigens, hence contributing in different ways to the bacillus recognition, phagocytosis, and to immune responses modulation. However, Mtb envelope components are not only encountered at the bacillus-host direct contact but can act remotely from the bacillus envelope. Indeed, they are also released from the bacillus envelope and are detected in different compartments such as the infected cells endosomal compartments or in extracellular vesicles produced by the bacillus itself or by infected cells. Characterizing their trafficking is of main importance for our understanding of their role in host-pathogen interactions and consequently for their potential use as vaccine components. This review aims at providing an overview of the current knowledge of the nature of Mtb envelope components shuttled within extracellular vesicles, the interaction of these vesicles with host immune cells and the remaining black holes.

## Introduction

Nowadays tuberculosis (TB) remains one of the top 10 causes of death worldwide with an estimated 1.4 million deaths in 2018 (1). The causative agent, *Mycobacterium tuberculosis* (Mtb), is an airborne pathogen that infects alveolar macrophages and dendritic cells primarily. The bacillus recognition by innate immunity receptors induces its phagocytosis and the initiation of the inflammatory response. Mtb has evolved strategies to adapt to the host intracellular environment and to modulate bactericidal activity of phagocytes to avoid killing, such by inhibiting the phagosome acidification and fusion with lysosome (2, 3). However, macrophage production of chemokines and pro-inflammatory cytokines (IL-12, IL-1β, and TNF) induces the recruitment at the infection site of different cellular populations, including T lymphocytes, that form a characteristic granuloma restraining the bacillus (4). The bacillus has also evolved evasion mechanisms to delay the onset of this adaptative immunity by modifying the balance of pro- and anti-inflammatory immune responses, in part by inducing the production of the anti-inflammatory cytokines IL-10 and TGF-β (5–8). Nevertheless, the presentation of bacterial antigens by dendritic cells in the lymph nodes leads to the recruitment of T cells with different effector properties, which also contribute in balancing the inflammation in the granuloma. This includes T_H_1 CD4^+^ T cells, which play an important role by producing IFN-γ that synergize with TNF in activating antimicrobial functions of macrophages like the inducible nitric oxide synthase (iNOS), phagosomal maturation or autophagy, and in maintaining the granuloma (9–12).

Finally, an arm wrestling match establishes between the host defenses and Mtb's evasion strategies. In 90% cases of infection, the bacillus is restrained in a latent state rather than killed. A better understanding of the intimate interaction between the host and the pathogen and the identification of immune correlates of protection are today needed to design new and effective anti-TB vaccine strategies (13).

Several fatty acid-containing components of the bacillus envelope, such as lipoproteins, lipoglycans, lipids, and glycolipids, are key players in these interactions (14–17). As modulators of both innate and adaptative arms of the immune response, they represent interesting candidates to integrate the formulation of new sub-unit vaccines against TB. The selection of the best candidates would intimately rely on a deep understanding of their biological properties and contribution in the orchestration of immune responses. The role of the bacillus envelope components in TB infection is a lot conceived within the envelope at the direct contact between the bacillus and the host cells. However, a new aspect of the biology of these components has emerged with the discovery that they traffic within the infected cells and within extracellular vesicles produced by the bacillus itself and by infected cells, here called bacterial membrane vesicles (BMV) and host extracellular vesicles (HEV), respectively. Both types of vesicles display important immunomodulatory properties on host cells. The characterization of the composition of these vesicles in Mtb envelope's components remains incomplete, especially regarding Mtb lipids and glycolipids. However, this trafficking might represent a key process by which bacterial factors contribute to host-pathogen interactions and TB pathogenesis, thank to long-distance effect beyond the infected cells. Indeed, the macrophage initially infected by Mtb being severally disabled, the modulation of potent non-infected cells is decisive for the outcome of the infection. Bacterial components of vesicles have the potential to favor or, in contrary, to impair the control of the infection, depending on their immunomodulatory properties. Such transfer of bacterial material could explain how only few bacteria can have such an intense influence on host tissue at the infection site, as within the granuloma.

During Mtb infection, BMV and HEV are also released. Not unexpectedly, their characterization has progressively highlighted that they shuttle diverse mycobacterial PAMPs and antigens and regulate host immune responses (**Figure 2**). The current manuscript aims at providing an overview of the described immunomodulatory properties of these vesicles, knowledge of the repertoire of mycobacterial factors they shuttled and how this trafficking extends our understanding of the role of Mtb envelope components in host-pathogen interactions, beyond the infected cells.

## Lipoproteins, Lipoglycans, and (Glyco)Lipids of Mycobacteria's Envelope Display Various Immunomodulatory Properties

**Figure 1:**
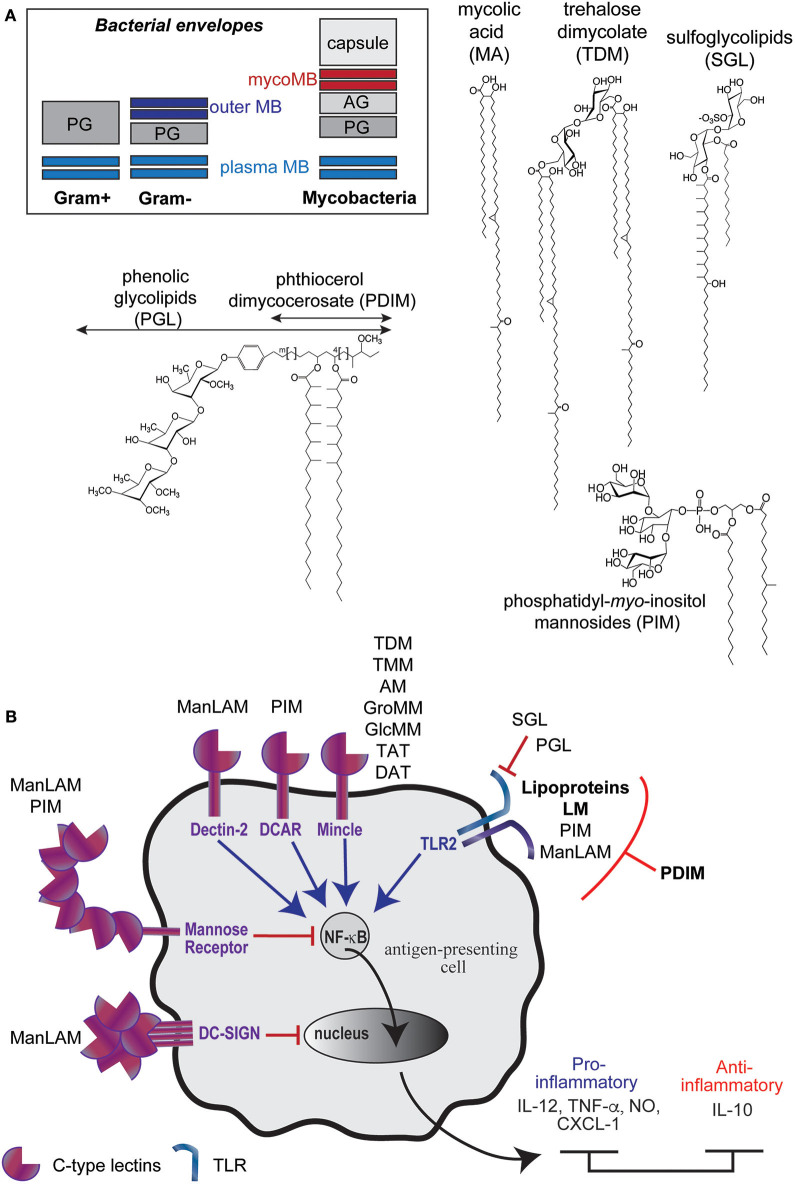
M. tuberculosis envelope encloses diverse immunomodulatory lipid and glycolipid families. **(A)** Schematic representations of bacteria envelopes (MB, membrane; PG, peptidoglycan; AG, arabinogalactan) and examples of unique (glyco)lipid structures found in mycobacteria envelope. **(B)** Most of Mtb envelope components have immunomodulatory properties, in particular though their interaction with Pattern Recognition Receptors.

Mycobacteria are characterized by a thick and protective envelope composed of different layers; an inner plasma membrane and an outer membrane (or mycomembrane), separated by the periplasm, the peptidoglycan and the arabinogalactan (18, 19) (Figure 1A). This envelope encloses lipoproteins, lipoglycans, and dozens of lipid and glycolpid families that distribute differently within the plasma membrane and the mycomembrane (20, 21). The plasma membrane is mainly composed of phospholipids, phosphatidyl-*myo*-inositol di-, and hexa-mannosylated (PIM_2_ and PIM_6_), triglycerides (TAG), lipoproteins such as LprG and LppX, and lipoarabinomannan (LAM). The mycomembrane is made of an inner leaflet composed of the characteristic mycolic acids (MA) (up to 90 carbon-long fatty acids) that are covalently bound to the arabinogalactan, itself linked to the peptidoglycan. The outer leaflet of the mycomembrane encloses, besides the LAM and lipoproteins such as LpqH, an impressive diversity of unique lipid and glycolipid families (Figure 1A). Among the envelope (glyco)lipidic components, mycolyl esters such as trehalose mono- and dimycolates (TMM and TDM) are found for all mycobacteria. In contrast, phthiocerol dimycocerosate (PDIM), phenolic glycolipids (PGL) or sulfoglycolipids (SGL) are strain-specific (glyco)lipid families.

Many of them have potent immuno-stimulatory or -suppressing properties on host cells by acting as (i) Pathogen-Associated Molecular Patterns (PAMPs) recognized by the Pathogen Recognition Receptors (PRRs) such as the Toll-Like Receptors (TLR) or the C-type lectins (DC-SIGN, Mincle, Dectin-2, DCAR), (ii) as antigens presented to unconventional CD1-restriced T cells or (iii) as virulence factors. By interacting with PRR, Mtb envelope components contribute to the bacillus recognition, which is followed by its phagocytosis and differential modulation of the inflammatory response (Figure 1B). For instance, the lipomannan (LM) (22, 23), the mycobacterial lipoproteins such as LpqH (24) and, to a weaker extent, the phosphatidyl-*myo*-inositol mannosides (PIM) (25), induce the production of pro-inflammatory cytokines through their interaction with Toll-like receptor 2 (TLR2). The interaction of Mtb envelope components with different C-type lectin(-like) receptors also initiates the bacillus phagocytosis and subsequently modulates inflammation. The mannose-capped lipoarabinomannan (Man-LAM) modulates immune response through several phagocytes receptors. Its recognition by Dectin-2 (dendritic cell-associated C-type lectin-2) leads to the production of pro- and anti-inflammatory cytokines (IL-6, TNF-α, IL-10, IL-2) in dendritic cells and promotes T cell-mediated acquired immunity (26, 27). In contrast, its interaction with DC-SIGN (dendritic cell-specific intercellular adhesion molecule-3-grabbing non-integrin) is described to mediate immunosuppressive activities (27–29). The ManLAM also contributes to the inhibition of the phagosome-lysosome fusion and inhibits the production of IL-12 through its interaction with the mannose receptor (29, 30).

Besides the lipoglycans and the lipoproteins, most of the (glyco)lipids families of the envelope modulate immune responses as ligands of PRR and/or as T cells antigens. One of the most abundant mycolic acid ester, the trehalose dimycolate (TDM), which also participates in the phagosome maturation arrest (31, 32), triggers a pro-inflammatory response (IL-8, IL-6, CCL2-4) through its interaction with Mincle and induces granulomatogenesis (27, 33, 34). The biosynthesis intermediate of TDM, the trehalose monomycolate (TMM) has been less studied but also binds to Mincle and displays similar adjuvant and granulomatogenic properties (35, 36).

Many mycobacterial (glyco)lipids activate unconventional T cells through their presentation by the CD1 proteins (CD1a, CD1b, and CD1c isoforms) (16). This includes mycolic acids and mycolyl esters (glucose/glycerol monomycolate), diacylated sulfoglycolipids, and PIM, which are presented by the CD1b isoform. CD1-restricted T cells are activated during the course of Mtb infection. They display cytotoxic functions or secrete pro-inflammatory cytokines that promote macrophage bactericidal activity. Evidences support that they contribute to the control of mycobacteria infection (37). Presentation of mycobacterial glycolipids requires accessory proteins that act as lipid tranfer proteins that assist antigen processing and loading onto transmembrane CD1 isoforms. The CD1e protein, unique soluble CD1 isoform, has been shown to assist the enzymatic hydrolysis of PIM_6_ into antigenic PIM_2_ in late endosomal compartiments and their subsequent loading in the CD1b isoform hydrophobic channels (38–40).

Other (glyco)lipid families mediate evasion from the macrophage microbicidal functions. For instance, the phthiocerol dimycocerosates (PDIM) virulence factors have been proposed to induce phagosome damage by inserting into host membrane and to alter TLR2 detection, and subsequent bactericidal and immune response, by masking cell wall exposed Mtb PAMPs (7, 41). The sulfoglycolipids (SGL) act through a different mechanisms, as a TLR2 antagonist (42), and have been recently shown to play intricate role with PDIM in inhibiting autophagy (43). Finally, phenolic glycolipids (PGL), which are produced by hypervirulent strains (44), would exploit the lectin receptor CR3 to promote bacterial uptake and to inhibit TNF-α secretion by human macrophages (45).

## Types of Extracellular Vesicles Released During Mycobacterial Infection

Vesicles are produced in all three domains of life under physiological and pathological conditions. They shuttle various types of cargos including cytoplasmic/cytosolic and membrane-associated proteins, lipids, glycoconjugates, and nucleic acids. Vesicles release is, besides cell-cell contact and exchange of soluble mediators, an additional mechanism of intercellular communication leading to the regulation of a plethora of functions in recipient cells. During Mtb infection, vesicles are released by the bacillus itself and by infected cells.

### The Release of Bacterial Membrane Vesicles (BMV) by Mycobacteria

It is today known that Gram-positive and Gram-negative bacteria release 20–400 nm vesicles, which are involved in cell physiology, such as stress response and detoxification, as well as in host-pathogen interactions and pathogenicity by shuttling PAMPs, toxins, or virulence factors (46). For Gram-negative bacteria or diderm bacteria, vesicles are released by a blebbing and pinching-off process from the LPS-containing outer membrane, therefore referred to as outer-membrane vesicles (47). However, outer-inner vesicles containing both cytoplasmic and periplasmic components have also been described to be produced by diderm bacteria (48). Although doubted for a long time, Gram-positive bacteria also produce vesicles, which release from the plasma membrane toward the extracellular space would involve cell wall-degrading enzymes that destabilize the peptidoglycan layer (49).

First described for *M. ulcerans* in 2007 (50), the vesiculogenesis has been next observed for all tested mycobacteria including Mtb and *M. bovis* BCG (51). First, observed for *in vitro* cultures, vesicles were later described in the phagosomes of infected macrophages (51, 52). Given the difficulty to isolate BMV from the phagosome of infected cells, the composition and properties of mycobacterial BMV have been essentially studied using vesicles isolated from broth culture supernatants. These vesicles cannot be assigned to artifact lipid aggregates or debris that would come from cell lysis for the reasons that their *in vitro* release requires bacillus viability and, although constitutive, is a regulated process. Indeed, in low iron medium, a challenging deprivation encountered within host cells, BMV production is stimulated and their content is enriched in the cell-associated mycobactin siderophore to support the essential needs in iron of neighboring bacteria (53). The release of vesicles is therefore a physiological process used by the bacillus to adapt to environment clues, notably by modifying the composition of these vesicles. In addition, vesiculogenesis regulators working independently have been highlighted to date: virR (vesiculogenesis and immune response regulator) (54) and the SenX3-RegX3 two-component system (55). The deletion of the first one or the activation of the second one leads to a hypervesiculation phenotype. The mechanisms of action of these regulators are still poorly understood because the characterization of the vesiculogenesis mechanism itself is still in its infancy in mycobacteria. Their high content in cytoplasmic proteins, lipoproteins, and phospholipids, which are also abundant components of the plasma membrane, supports the hypothesis that BMV originate from the plasma membrane of the mycobacterial envelope. How these vesicles traffic across the above layers of the envelope to reach the extracellular space is a question widely opened (Figure 1A). As for Gram-negative bacteria it might include the action of degrading enzymes. In this sense, in a recent preprint article the groups of M. Rodriguez and R. Prados-Rosales are describing that vesiculogenesis is accompanied of peptidoglycan alterations and that the dynamin like proteins IniA and IniC are required for Mtb-EV biogenesis in culture and during macrophage infection (56).

### The Release of Host Extracellular Vesicles (HEV) by Mycobacteria-Infected Cells

Eukaryotic cells release three main types of vesicles classified based upon their biogenesis, release pathway, size, and content: exosomes (30–150 nm) are released following the fusion of multivesicular endosomes (MVE) with the plasma membrane and are characterized as tetraspanins positive vesicles (CD9, CD63, CD81), microvesicles (100 nm−1 micron) originate from the outward budding of the plasma membrane and carry selectins and integrins at their surface and apoptotic bodies (1–5 microns) result from apoptotic cells disassembling and can be differentiated by the presence of histones and cellular organelles (57). Hence, the different types of vesicles can be purified from cell culture supernatant or biological fluids based on their physical properties. The most commonly used protocols for the separation of these main types of vesicles consist of differential centrifugation allowing the successive sedimentation of apoptotic bodies (~2,000 g), microvesicles (~10,000 g) and the smallest vesicles such as exosomes (~100,000 g). The centrifugation if often followed by an extra purification step on density gradient to remove co-precipitated HEV-associated proteins and nucleic acids (58). However, progress in the field of vesicles purification and characterization keep highlighting the existence of multiple sub-populations among these main types of vesicles with overlapping in size and markers, which add a level of complexity for their individual separation and functional characterization (59).

**Figure 2:**
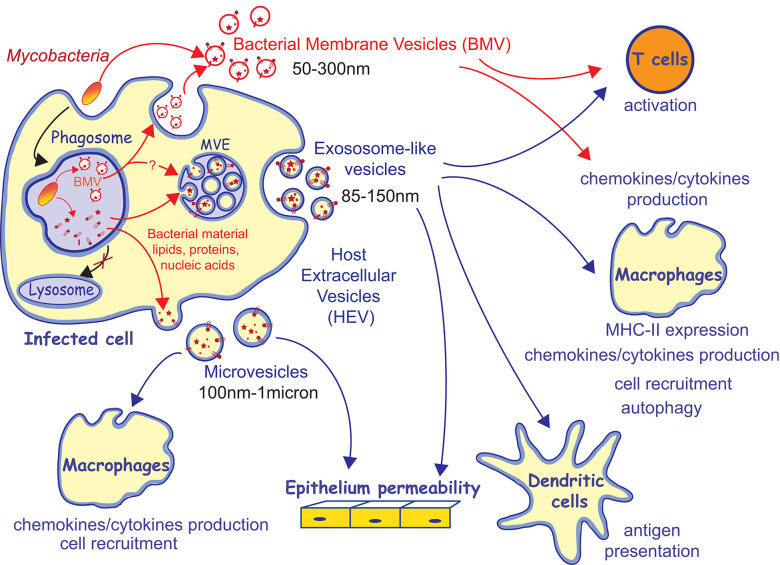
Trafficking of *M. tuberculosis* factors within vesicles and their immunomodulatory properties. Mycobacteria release bacterial membrane vesicles (BMV) in broth culture and within infected cells. It has been proposed that BMV are released from infected cells through exocytosis. Bacterial material released from the mycobacterial phagosome traffics toward multivesicular endosomes (MVE) and the plasma membrane and is released within host extracellular vesicles (HEV), including exosomes and microvesicles. BMV and HEV containing mycobacterial material are capable to modulate many arms of the host immune response.

As every eukaryotic cells, macrophages infected by mycobacteria release exosomes, microvesicles, and apoptotic bodies. Although their content in mycobacterial factors is currently characterized to different extent, all these vesicles are endowed with important immunomodulatory properties (Figure 2).

## Molecular Characterization of Mycobacterial Membrane Vesicles

The (glyco)lipid content of BMV released by the ^14^C-labeled BCG or Mtb strains was analyzed by 2D-TLC (51). The analysis of retention factors highlighted a high abundance of PIM_2_Ac_2_, PIM_2_Ac_1_, and phospholipids (PG, PE, PI, and cardiolipin) and lower amount of polyacylated trehalose and phenolic glycolipids but several lipids remained unassigned. In addition, MALDI-Tof analysis of Mtb and BCG BMV extracts has also identified PIM_6_Ac_1_. The high abundance of plasma membrane lipids supports the hypothesis that BMV originate from the inner membrane of mycobacteria envelope.

Besides lipids and glycolipids, proteomic analyses of the BMV released by Mtb and *M. bovis* BCG, have identified hundreds of mycobacterial proteins including virulence-associated proteins and immunodominant antigens (superoxide dismutase, catalase, Ag85, and CFP10) and have revealed an enrichment in lipoproteins (LprG, LpqH, LprA, PstS1, and LppX), which are strong TLR2 agonists (51, 60). Interestingly, this enrichment in lipoproteins was not observed for the BMV released by the avirulent strain *M. smegmatis*, suggesting that BMV composition and therefore subsequent biological properties, differ between mycobacterial strains.

## *In vivo* and *in vitro* Immunomodulatory Properties of BMV Released by Mycobacteria

Mycobacteria BMV are capable to regulate both innate and adaptative immune responses *in vitro*. Mouse bone marrow-derived macrophages (BMM) stimulated with purified BCG BMV produce IL-1β, IL-6, IL-12, TNF, CXCL1 in a TLR2-dependent way as well as MIP-1α and IL-10, independently of TLR2 (51, 52). In addition, BMV are capable to deliver antigens to dendritic cells leading to T cells stimulation (61). In contrast, Mtb BMV were described as inhibiting CD4^+^ T cells activation *in vitro* (62) through the direct delivery of LAM, which has been previously shown to repress TCR signaling (63).

Biological properties of mycobacterial BMV have also been evaluated *in vivo*. The intra-tracheal administration of BCG BMV to mice prior to Mtb infection, leads to a local exacerbation of the disease with an alteration of the pulmonary myeloid populations, an increased granulomatous inflammation in the lungs and higher bacterial loads in lungs and spleens (51, 60). BMV released by the avirulent *M. smegmatis* strains, which are not enriched in lipoproteins, do not induce such intense inflammation. In contrast, the subcutaneous immunization of mice with Mtb BMV induces humoral response and a strong splenic Th1 cellular response that is directed against cell wall and plasma membrane components of the bacillus but essentially against lipoproteins. Finally, this immunization allowed the control of Mtb replication in lungs, as efficiently as the BCG vaccination (64). The contradiction between these two *in vivo* studies regarding the beneficial or deleterious impact of BMV immunization on the infection control can be explained by differences in immunization protocols. Notably, the vesicles are not inoculated through the same route and the first study used doses of BMV 10 times higher, which may explain the exacerbated inflammation.

## Mycobacterial Components Shuttled by HEV Released by Infected Cells

### Exosome-Like Vesicles

Among HEV released by mycobacteria-infected cells, exososome-like vesicles are the most characterized category of vesicles, at the molecular and functional levels, essentially due to chronological reasons. The release of mycobacterial material from the phagosome, where the bacillus resides, and its intracellular trafficking within host cells was first described by the laboratory of D. Russell in 1994 for *M. avium* and later extended to other mycobacterial species (65–69). Using global radioactive or fluorescent labeling of the surface compounds of Mtb H37Rv, Mtb H37Ra, *M. bovis* BCG (BCG), or *M. avium* strains, the authors described the release of mycobacterial material from the macrophage phagosome toward other intracellular compartments. As soon as 4 h post-infection, labeled bacterial material is detected in the perinuclear space, the Golgi network, and accumulate in late endosomes and lysosomes of the infected cells, including LAMP-1 and MHC class II positive MVE (Figure 2). Finally, as soon as 16 h post-infection, an estimated 12% of the labeled material initially associated to the bacillus are released within 100–200 nm extracellular vesicles. These vesicles were defined at the time as being exosomes because of their link with the MVE and because following studies highlighted exosomal host proteins like LAMP1, LAMP2, MHC-I, and MHC-II and tetraspanins (CD81, CD86, CD63, and CD9) (70, 71). According to the current state of the art, these criteria might not be sufficient to name these vesicles as *bona fide* exosomes, as stressed by the International Society of Extracellular Vesicles (72). However, for simplicity reason, the term “exosomes” is conserved thereafter to refer to the properties of the vesicles that sediment at high-speed centrifugation (100,000 g).

The mycobacterial components shuttled by these exosome-like vesicles have been investigated through several approaches. Immunogold electron microscopy experiments and western blot analyses have identified the PIM_2_, LAM, LM, and the LpqH (19kDa) and LprG (27kDa) lipoproteins as part of the material associated to the membrane of MVE and released in exosomes (65, 68). However, ^14^C acetate or fluorescein/biotin hydrazide-based labeling approaches globally target a wide number of envelope components including lipids, glycolipids, glycoproteins, lipoproteins, and lipoglycans. Bacterial (glyco)lipids present in MVE or in exosomes released by infected cells could not be harvested in sufficient quantity at the time to undertake their structural characterization. Nevertheless, their analysis by thin-layer chromatography (TLC)—autoradiography showed the presence of molecules with retention factors similar to the ones of trehalose dimycolate, trehalose monomycolate, PIM_2_Ac_2_, PIM_2_Ac_1_, monoglycosylated PGL (mycoside B), cardiolipin, and phosphatidylethanolamine besides many unassigned components (65, 67). The release of mycobacterial (glyco)lipids from the phagosome and their intracellular trafficking was further supported by Fisher et al., who also highlighted that cardiolipin is processed in lyso-cardiolipin by the lysosomal Phospholipase A2 during its trafficking (73).

Proteomic analyses were later performed on exosomes isolated from *in vitro* culture and from patients blood and have identified up to 41 mycobacterial proteins including highly immunogenic ones such as the antigen 85A, B and C, MPT64, and ESAT-6 (74, 75). Finally, exosomes released from Mtb-infected RAW264.7 macrophages or BMM were recently shown to shuttle several mycobacterial transcripts (76–78).

### Apoptotic Bodies and Microvesicles

The mycobacterial components of others type of HEV released by mycobacteria-infected macrophages have not been characterized in such details. Nevertheless, using macrophages infected by [^14^C] palmitic acid and [^14^C] amino acids labeled-*M. bovis* BCG, Schaible et al. described in 2003 by similar 2D TLC and western blot analyses that the 19kDa lipoprotein and trehalose dimycolate, mycoside B, lysocardiolipin, and PIM are shuttled by extracellular vesicles (79). The authors identified these vesicles as apoptotic bodies based on large diameter (>2 microns) and the absence of the late endosome/lysosome marker cathepsin D. MHC-II, CD1, and the mannose receptors were present at the surface of these vesicles. The bacterial components shuttled by the microvesicles released by Mtb-infected macrophages have not yet been deciphered although mycobacterial protein antigens are expected given the fact that microvesicles have the capacity to activate specific-T cells as detailed later.

## Trafficking of Mtb Envelope Components Within BMV and Within HEV - Not Such an Obvious Connection

There is today an important body of data showing that mycobacterial material exits the phagosome where the bacillus resides within infected cells. However, the different steps of this intracellular trafficking are not fully dissected including (i) the mechanisms responsible for the release/dissociation of mycobacterial components from the bacillus and (ii) the routes taken by these compounds and their intersection with the biogenesis pathways of the different extracellular vesicles (Figure 2). Although the release of BMV may appear to be the obvious source of mycobacterial compounds that feeds HEVs, their connection is currently not easy to establish. First, if the release of BMV requires bacillus viability, the detection of labeled mycobacterial surface compounds in the MVE, from which exosomes originate, does not (67). In addition, BMV and HEV likely release distinct Mtb factors. Indeed, the (glyco)lipid profiles of exosomes and BMV on TLC do not overlap. Currently available data are highlighting that BMV contain a predominance of mycobacterial plasma membrane lipids as compared to cell wall lipids, while exosomes profiles show a wider diversity of (glyco)lipids including several families of the mycomembrane (52, 67). It is likely that parallel mechanisms of release from the bacillus coexist. In parallel to the release of BMV, uncovalently bound surface components might also shed from the bacillus envelope, naturally or as the result of the envelope degradation in the enzymatic and mildly acidic environment in the phagosome but this still needs to be demonstrated. Once released from the bacillus, the mechanisms by which Mtb components leave the phagosome to intercept the HEV biosynthesis pathways is not understood. Recycling event from the phagosome and membrane exchange with endosomes might contribute in addressing mycobacterial envelope components toward the plasma membrane and endosomal compartments, from which microvesicles and exosomes originate, respectively (80–82). In addition, it has been described that mycobacteria are capable to damage the phagosomal membrane, which can also contribute to the leakage of mycobacterial molecules outside of the phagosome (83, 84).

Therefore, BMV, exosomes, and microvesicles might shuttle different repertoires of Mtb immunomodulatory molecules. In addition, vesicles composition in mycobacterial cargos is dynamic and evolves over the course of Mtb infection. Indeed, the capacity of exosomes, derived from cell cultures or from biological fluids, to induce cytokines/chemokines production depends on the time they were collected post-infection (71). In the same vein, initial work performed by the laboratory of D. Russell described that labeled mycobacterial lipidic material is released from the phagosome and detected in exosomes as soon as time as 16 h post-infection but the LAM and the LprG lipoprotein are only detected in the pellet of exosome-like vesicles after 48 h post-infection (66, 85). The recent work of Athman et al. also illustrated that small vesicles of varying composition co-exist. Studying vesicles released from RAW264.7 cells 24 h post-infection, authors described that the pool of the so called “exosomes” sedimented at 100,000 g and previously described as containing mycobacterial envelope components encloses two vesicle sub-populations that can be separated on continuous sucrose gradient, (i) vesicles carrying the LAM and LM but devoid of ones of the exosomal markers CD9 and CD63 and inversely (ii) vesicles bearing the CD9, CD63, and CMH-II host proteins but devoid of LAM and LM (52). The detection of the LAM and LM positive vesicles was dependent on the bacillus viability. Hence, Athman et al. proposed that the LAM and LM-containing vesicle sub-population are BMV produced by the bacillus and subsequently exocytosed into the extracellular environment, rather than exosomes (Figure 2). The second LAM and LM-negative vesicles may also correspond to a sub-population of HEV that shuttle different mycobacterial components. Indeed, the limits between the main types of vesicles released by eukaryotic cells is becoming thinner due to overlap in size, density, and even in markers and vesicles subpopulations appeared to be more and more diverse (59). Indeed, different subpopulations co-purifying with *bona fide* exosomes have been described, including CD9^−^CD63^−^ vesicles (86).

## Modulation of Innate Immune Responses by HEV Released by Mycobacteria-Infected Cells

The possible internalization of exosomes by neighboring non-infected cells *in vitro* garnered significant interest into the characterization of the immunomodulatory potential of these vesicles in the following years, in which the laboratory of J. S. Schorey had a major contribution. Most of the studies were performed using the so called exosomes purified from the supernatants of *in vitro* cultures of monocytic cells infected by *M. avium*, BCG, or H37Rv strains through differential centrifugation, by sedimentation at 100,000 g, combined to sucrose density gradient (70, 71, 85, 87, 88). However, exosomes purified from mice biological fluids or released by human neutrophils (89) have also been studied. Overall, isolated from *in vitro* cultures of infected cell lines or from biological fluids of infected animals, exosomes are capable to induce a general pro-inflammatory response in naïve macrophages *in vitro* and *in vivo*. Indeed, exosomes released by mice or human monocytic cell lines infected by Mtb H37Rv or BCG induce the iNOS expression and several cytokines and chemokines production *in vitro* including TNF-α, RANTES, MIP−1α, MIP−1β, MIP−2, and IL1-Ra (68, 70, 71). The induction of TNF-α production is completely dependent on MyD88 and partially but significantly dependent on TLR2 or TLR4. These properties of exosomes are likely due to the presence of bacterial factors rather than host mediators produced by activated macrophages because exosomes released by macrophages activated by the LPS or infected by heat-killed mycobacteria failed to induce the same responses (85). Similarly, exosomes released by Mtb infected-human neutrophils contain TLR2/6 ligands and strongly induce the production of TNF-α, IL-6, superoxide anion and the expression of co-stimulatory molecules (CD80, CD86) by autologous macrophages (89). Like cell culture–derived vesicles, exosomes isolated from biological fluids (BALF or the serum of BCG-infected mice) promote a pro-inflammatory response of BMM such as the production of cytokines and chemokines, including TNF-α, RANTES MIP−1β, MIP−2, IL1-Ra, and IL-27 (71, 85). Exosomes derived from Mtb-infected cells or from infected mice serum also stimulate inflammation of endothelial cells by increasing monolayers permeability and inducing the expression of TLR2, VCAM1, and CCL2 (90). Exosome-induced pro-inflammatory response has also been described *in vivo*. Indeed, the intranasal injection of exosomes released by H37Rv- or BCG-infected THP-1 cells leads to a robust production of IL-12p40 and TNF-α and the increase of neutrophils in the BALF and lungs of BALB/c mice, respectively (71, 85). The treatment of infected mice with a combination of moxifloxacin and vesicles released by H37Rv-infected BMM also leads to higher serum levels of TNF-α, IL1-β and IFN-β, the later one being dependent on the MAVS RNA-sensing pathway.

Exosomes containing Mtb material also modulate the microbicidal potential of infected-cells and have a positive effect on the overall control of the infection. Exosomes released by Mtb-infected BMM or neutrophils are capable to promote autophagy, which is a key mechanism of intracellular pathogen clearance (77, 89). Indeed, treatment of BMM with a combination of IFN-γ and Mtb-infected cell exosomes leads to an increased colocalisation of bacteria with LC3 or LAMP-1 positive compartments, in a RIGI/MAVS RNA sensing pathway dependent manner (77). The induction of Mtb-containing phagosome maturation contributes, in part, to the reduced intracellular survival of Mtb observed when host cells are treated by Mtb-infected cell exosomes (77, 89).

The functional characterization of microvesicles released by Mtb-infected cells highlighted *in vitro* and *in vivo* similar immunomodulatory properties as for exosomes, including enhanced cytokines, and chemokines production, permeability of epithelial monolayers and neutrophils and dendritic cells recruitment (91).

## HEV Released by Mycobacteria-Infected Cells Modulate Adaptative Immunity

HEV released by Mtb-infected cells have been shown to modulate adaptative immune response in 2 ways, by regulating (i) the activation of antigen-presenting cells (APC) and (ii) the stimulation of T cells.

It has been reported that exosomes released by Mtb-infected macrophages display immunosuppressive properties on the activation of antigen-presenting cells. Indeed, they suppress the IFN-γ mediated activation of naïve BMM by inhibiting the expression of MHC class II and CD64 in a TLR2- and MyD88-dependent way (88). However, exosomes containing Mtb components promote the activation of antigen-specific MHC-restricted CD4^+^ and CD8^+^ T cells *in vitro* and *in vivo* (87, 92, 93). Microvesicles derived from Mtb-infected BBM are also capable to stimulate Ag85 specific-T cells (93). Interestingly, dendritic cell-derived exosomes bearing MHC and co-stimulatory molecules as well as MHC-II positive microvesicles are capable of direct *in vitro* activation of T cells in absence of APC (87, 92, 93). Finally, the intranasal administration of exosomes released from macrophages treated with BCG culture filtrate proteins (CFP) have been shown to prime a protective immune response and to boost a prior vaccination in mice, with a protecting effect that is at least as good as the BCG vaccine (94).

Activation of MHC- and CD1 (a,b and c)-restricted T cells was also demonstrated to be mediated by the apoptotic bodies released by Mtb-infected macrophages *in vitro* and *in vivo* (79, 95). However, it is to note that if the exosome and microvesicle release is enhanced during mycobacterial infection (85, 93) it has been described that Mtb has developed strategies to evade apoptosis (96, 97). Finally, if exosomes shuttle antigens of CD1-restricted T cells such as PIM, their capacity to activate CD1-restricted T cells has not been tested so far.

While Mtb is sequestered in the phagosome of host macrophages and inhibits its maturation, it isolates itself from the antigen processing and presentation pathways necessary for the activation of T cells, which play a key role in infection control. By shuttling mycobacterial factors outside of the disabled infected cells, the vesicle release therefore provides an alternative pathway to activate MHC- and likely CD1-restricted T cells by uninfected bystander APC, also called cross-presentation.

## Biological Relevance and Benefits of Mtb Envelope Components Release Within Vesicles for The Control of Mtb Infection

There is no longer any doubt that during infection, cytosolic and envelope components of Mtb travel within BMV and HEV between the bacillus, the infected cells and the uninfected bystander cells, impacting both the innate and adaptive immune responses. However, it is still difficult to say whether the release of mycobacterial compounds through vesiculogenesis benefits the host or the bacillus because several important questions remain opened. The truth is probably halfway since vesicles have shown properties that go both ways. If the protective effect of immunization assays performed with purified BMV or HEV are supporting beneficial effects of vesicles release, these assays do not reflect the heterogeneity of vesicles encountered during Mtb infection, which is not fully deciphered and turns out to be more and more complex according to recent studies (52).

One way to evaluate the impact of a biological process is to repress it. However, it would be necessary to characterize the different populations of released vesicles and to gain knowledge of their biosynthetic pathways. If our understanding of the vesiculogenesis mechanisms in Mtb is at its premise, the biogenesis pathways of eukaryotic vesicles were revealed to be diverse and complex (57). For instance, exosome biogenesis and release utilize several machineries depending on cell type or physiological status. In addition, repressing vesiculogenesis is a debatable strategy because vesiculogenesis shares pathways with essential cellular processes like cellular membrane trafficking and secretion. Hence, its repression might lead to additional adverse effects on the overall physiology of the cell. Nevertheless, Smith et al. have tested such approach by studying the trafficking of mycobacterial peptide antigens within exosomes and subsequent T cells activation, in mice that are deficient in Rab27a (92), a GTPase involved in the MVE docking to the plasma membrane during exosomes release (98). Authors observed in this genetic background a diminished trafficking of extracellular mycobacterial proteins, a reduced concentration of exosomes in mice serum and a reduced T cell activation that is associated to an increase bacterial burden. This study further supports the beneficial effect of exosomes release in the control of Mtb infection. Similarly, future investigation of the phenotype of Mtb strains deficient for the IniA and IniC dynamins recently identified as involved in BMV biogenesis (preprint article of Gupta et al.) (56) would provide insights into the effect of BMV release on bacterial recognition vs. evasion and whether dynamin-like proteins can be candidate target for the development of new anti-TB therapeutic strategies.

## Potential of Extracellular Vesicles as Anti-TB Vaccine and Biomarkers

Extracellular vesicles content reflects the physiological state of the releasing cells by shuttling microbial PAMPs or tumor antigens. From microbes or eukaryotic cells, released vesicles detected in biological fluids, and capable to modulate immune responses, have been proposed as platforms for the development of diagnostic or vaccine tools in different fields (99, 100). Same perspectives can be transposed in the Tuberculosis field.

Indeed, exosomes are detected in human and mice biological fluids during mycobacterial infection, more than 100 days post infection (101). Their abundance is shown to correlate with bacterial burden and proteomic analyses have identified mycobacterial protein components that are consistently detected in patients' serum (75). Therefore, detection of exosomal mycobacterial proteins show great promise for the development of diagnostic test, which could be further reinforced by the detection of mycobacterial envelope components and transcript cargos as well as host factors.

Given their adjuvant and antigenic properties described above, BMV and exosomes may also appear as interesting vaccine candidates. However, moving from animal immunization assays to clinical trials still requires further characterization of the different vesicles subpopulations and of their composition. In particular, the current characterization of the exosomes and BMV content in Mtb envelope compounds remains based on conventional biochemical approaches of moderate sensitivity. The use of latest-generation methods would make possible to establish a finer composition of these vesicles especially regarding (glyco)lipids described as virulence factors, which hold risks of provoking toxic adverse effects.

Lastly, besides incomplete molecular characterization and the potential problem of non-autologous antigens for HEV, the use of native vesicles in clinical trials might face the technical challenge of the reproducibility of vesicles preparations as previously reported (64). Indeed, previous studies have shown that their composition in mycobacterial components and in protein involved in antigen presentation such as MHC molecules, depend on the releasing-cell type, the time of collection related to infection or the purity of vesicles, which are protocol- and laboratory-dependent.

Nevertheless, knowledge coming from the molecular and functional characterization of BMV and HEV populations can inspire the development of vesicle-based vaccine formulation. It would consist in tailoring vesicles with selected immunomodulatory components to induce an optimized protection. The use of vesicle-based approaches have gained interest as delivery system for the development of therapeutic and vaccine strategies in different pathological contexts (102). Vesicle-based delivery system of bioactive molecules present several advantages: (i) to allow the simultaneous delivery of molecules of various nature in a concentrated and protective way, (ii) to have the capability to overcome natural barriers, (iii) to display natural cell-targeting abilities, (iv) to provide a physiological membrane environment that is important for membrane protein stability as well as for the biological activities of Mtb (glyco)lipids. Indeed, it has been described that *in vitro* immunomodulatory properties of Mtb (glyco)lipids differ with the way they are formulated. For instance, differences in the induction of cytokines release have been observed whether the lipids are resuspended in solution or coated on beads of different size (35, 103). Such differences might also exist when shuttled within different vesicle sub-populations.

## Conclusion and Future Directions

Our knowledge of the released populations of vesicles and their immunomodulatory properties during Mtb infection has enormously progressed over the last 15 years. Vesicles study has highlighted the large diffusion, beyond the infected cell, of Mtb envelope components already known to have important immunomodulatory properties. Among various other cargos, Mtb envelope lipoproteins, lipoglycans, and (glyco)lipids certainly contribute to vesicles immunomodulatory properties in particular in the regulation to the inflammation process. However, direct connections need to be demonstrated, which could be achieved by analyzing the properties of vesicles released during infection by biosynthesis mutant Mtb strains (if available).

Further, progress in our understanding of the role of, both, vesicles and immunomodulatory components of Mtb envelope, in host-pathogen interactions and in the outcome of the infection will require a better characterization of (i) the diversity of the subpopulations of released HEV, (ii) the content of BMV and HEV in immunomodulatory mycobacterial (glyco)lipids and (iii) the variation of vesicles composition depending on the encountered mycobacteria strains and on the releasing-cell type. It would also be of great relevance to perform detailed molecular investigations on vesicles that better reflect the subpopulations encountered *in vivo*, working with biological fluids, which is today conceivable thanks to the high sensitivity of all the “omic” methodologies. In addition, means to regulate the biogenesis of BMV and HEV will allow determining the overall beneficial or deleterious effect of vesicles release during Mtb infection.

Finally, research on vesicles released during tuberculosis infection will likely inspire future development of new diagnostic tools and cell-free vesicle/liposome-based vaccine strategies.

## Author Contributions

EL wrote the manuscript.

## Conflict of Interest

The author declares that the research was conducted in the absence of any commercial or financial relationships that could be construed as a potential conflict of interest.

## Abbreviations

APC: antigen-presenting cell
BALF: Bronchoalveolar fluid
BMM: mouse bone marrow-derived macrophages
BMV: bacterial membrane vesicles
HEV: host extracellular vesicles
PAMP: Pathogen Associated Molecular Patterns
PRR: Pathogen Recognition Receptors
RIGI/MAVS: retinoic acid inducible gene-I (RIG-I)–mitochondrial antiviral-signaling protein (MAVS).
